# Assessing the 1918/19 Pandemic Influenza and Respiratory Tuberculosis Interaction in Malta: Operationalizing a Syndemic During a Crisis Event

**DOI:** 10.3390/tropicalmed10060149

**Published:** 2025-05-24

**Authors:** Lianne Tripp, Larry A. Sawchuk, Charles J. Farrugia

**Affiliations:** 1 Department of Anthropology, Trent University, Peterborough, ON K9L 1Z8, Canada; 2 Department of Anthropology, University of Toronto Scarborough, Toronto, ON M1C 1A4, Canada; 3 Department of Library Information and Archive Sciences, University of Malta, MSD 2080 Msida, Malta; charles.j.farrugia@um.edu.mt

**Keywords:** syndemic, 1918/19 influenza, respiratory tuberculosis, harvesting effect, life expectancy, crisis events, temperature, relative humidity, rainfall, Malta

## Abstract

Studies have indicated that there was a disease interaction of pandemic influenza with respiratory tuberculosis (TB) in Malta, which could explain the heightened mortality recorded in young adults. We revisit the 1918/19 influenza and TB syndemic potential on the island of Malta. Borrowing from crisis studies that explore the harvesting effect, we used the method of assessing changes in pre-pandemic, pandemic, fallow, and post-pandemic mortality/life expectancy to reveal the syndemic experience. Pre-pandemic (1914–1917) life expectancy at birth was significantly higher, at 37.91 years, than during the pandemic (1918), when life expectancy dropped to 33.26 years (Z = 10.56, *p* < 0.0001). Post-pandemic (1919) life expectancy rose to 43.49 years, which was an even longer life expectancy than pre-pandemic (Z = 17.61, *p* < 0.0001). There were significant changes in TB mortality death rates during the four periods in those of reproductive age. Augmenting our framework for studies of syndemics involving short-term events, we proposed the identification of contributing, driving, and limiting factors. Underlying living conditions contributed to the syndemic. The exacerbation of housing conditions, the economy associated with the First World War, and meteorological measures—temperature, relative humidity, and rainfall—were driving factors. The early implementation of mitigation strategies, such as restrictions on mass gatherings, were limiting factors of the syndemic.

## 1. Introduction

Considerable attention has been directed at the interaction of respiratory tuberculosis with 1918/19 pandemic influenza, which had the potential to result in more than expected deaths from influenza, or from the common secondary infection of pneumonia [1,2,3,4]. It is believed that the biological synergism of these diseases resulted from the activation of latent *Mycobacterium tuberculosis* or the acceleration of the progression of illness by the influenza virus [5]. Tuberculosis increased the likelihood of mortality from influenza [2], particularly in people who acquired secondary bacterial pneumonia. Due to the potential synergistic interaction between tuberculosis and the 1918/19 pandemic influenza, which may have manifested more frequently among the impoverished and marginalized, some scholars have characterized this relationship as a syndemic [4,6,7]. In Mamelund’s and Dimka’s (2019) study [1], they found a significantly higher morbidity rate of influenza-like illness (20.6%; *p* = 0.002) in females who had respiratory tuberculosis relative to employees in two sanitoria in Norway [1]. Noymer’s and Garenne’s (2000) study of the USA found that males infection with pandemic influenza had a higher age-standardized death rate from respiratory tuberculosis at 176 per 100,000, and that post-pandemic tuberculosis death rates fell to 107 per 1000, along with there being a temporary increase in life expectancy [2]. In 2009, Noymer found support for passive selection in their study on Union Army veterans; there was a significant effect of TB infection in the company on influenza deaths (hazard ratio= 1.098–1.118, *p* = 0.037–0.018 depending on model), but there was not a significant increase in influenza mortality among those with TB [3]. Tripp, Sawchuk, and Saliba, (2018), in addition to finding excess mortality from TB in Malta during the month of October 1918, also found monthly correlations of tuberculosis cases with influenza morbidity by town in Malta (Rho = 0.58, *p* = 0.001) [4].

The concept of syndemics was proposed by the medical anthropologists Dr. Merrill Singer and Dr. Charlene Snipes in 1992 (Singer had previously presented the term at American conferences in 1991). They recognized that the “synergistic nature of the health and social problems facing the poor and underserved” places those living in inner cities at greater risk of diseases such as AIDs [8] (p. 225). Fundamental to the syndemic concept is the importance of the social environment; in the case of AIDs, this was understood as *S*ubstance *A*buse and *V*iolence and *A*IDs, culminating in the SAVA syndemic [9].

One would expect that our history with diseases and other traumatic events would reveal syndemic occurrences. Singer [7] has proposed numerous examples from famines (such as the Irish famine of 1741) and migrations (such as the Mormons) to wars (such as World War One; see also [10]). For each of these syndemic events to arise, poor economic and social conditions, along with the interaction of malnourishment and infectious disease, drove mortality rates to unprecedented levels. It has also been proposed that potential historical examples of syndemics primarily involving infectious diseases include the following: scarlet fever in 1800s Massachusetts; the 1918/19–20 influenza pandemic around the world (tuberculosis and influenza); and the 1865 cholera epidemic in Gibraltar.

The veracity of these historical events as satisfying the features of a syndemic unfortunately cannot be supported with quantitative evidence, because in many cases it is too onerous or impossible to source reports and data to allow for the exploration of syndemics. Secondly, and more importantly, regardless of the period of the occurrence of the proposed syndemic, there has yet to be a consensus as to a method for “testing” the syndemic criteria or model. Mendenhall and Singer [11] point out an obvious reason for the lack of direction when evaluating a syndemic that is quite often overlooked by health researchers. Syndemic theory originated through ethnography but quickly pivoted to more epidemiological approaches for assessment; over time, numerous approaches have been proposed for the evaluation of syndemics. The diversity in quantitative methods for assessing a syndemic can vary extensively; for syndemic studies on HIV and AIDS alone, various statistical approaches have been utilized: regression analyses, higher-level modeling techniques, frequency and descriptive statistics, longitudinal cohort studies, and social network analyses [11].

At the population level, the comorbidity of two or more diseases or health conditions where the interaction of the diseases “exacerbates the negative health effects of any or all of the diseases involved” [12] (p. 941) is a syndemic, which is a concept that is akin to the ecological concept of synergy. Although the concept has arguably evolved over the last three decades, two constants remain: (1) “noxious social conditions” [9], such as social inequality, be it poverty, overcrowding, stress, or stigma, precipitate increased disease clustering, or physical or behavioral vulnerability [12] and (2), as a result of the synergistical interaction of two or more epidemics [or health problems], there is an “excess burden of disease in a population” [13] (p. 425). Due to the fact that a core element of the syndemic concept is deep-seated inequality, subpopulations that endure a high level of vulnerability are identified.

We borrow the concept of the harvesting effect from studies on crisis events. Otherwise known as forward mortality displacement, harvesting exhibits itself with a temporary increase in the number of deaths of weak, older persons or persons at risk of infection during periods of abnormal stress. Climatic extremes, hunger, bad quality of air, and epidemics can be prime factors in the harvesting effect [14,15,16,17]. Harvesting is followed by a period of a decrease in mortality rate, although temporary, in the age group that had been largely removed. The “healthy” or fallow period is another signifier of “excess burden”, because without a crisis event, there would not be a subsequent temporary period of enhanced survivorship.

We posit that the identification of a syndemic triggered by a short-term well-defined novel event can be revealed through the presence of the harvesting effect. The application of the harvesting effect for assessing the presence of a syndemic can offer the means for a quantifiable criterion of a syndemic—one that is replicable. Once the occurrence of a syndemic has been established, then the underlying social factors and or health problems contributing to the syndemic can be assessed.

This paper revisits the syndemic potential of 1918/19 influenza and respiratory tuberculosis on the island of Malta, which was reported by two of the authors (see [4]). Because the historical vital statistics for Malta provide death and population counts by age over a long time period, we can utilize a previously proposed methodology to operationalize the syndemic [18]). We use life table analysis to assess whether the *excess burden* of the syndemic was observable from a quantifiable means. This method incorporates the concept of the harvesting effect as a framework to evaluate pre-epidemic/pandemic or baseline mortality, epidemic mortality, and fallow and/or post-epidemic mortality to reveal significant changes in mortality and life expectancy.

We also explore the association of environmental factors such as temperature and relative humidity with the potential syndemic. The underlying social factors that contribute to, drive, and limit the syndemic are discussed. The social risk factors for 1918/19 influenza are debated among scholars. There is, however, an overall consensus that socio-demographic factors such as large population size, high population density, malnutrition, poverty, overcrowded living environments, low education levels, and urbanization, or contradictorily rurality, were contributors (to varying degrees) during epidemics of 1918/19 pandemic influenza, observed in many countries around the world [19,20,21].

### 1.1. Core Features of a Syndemic

One can argue that the syndemic approach has a greater potential to better capture and reveal the full range of complex interrelationships that exist in the epidemic experience than what has previously been used by health scholars. At the core of this theoretical construct are four fundamentals: disease clustering, social factors, and their interaction, which yields an excess burden that is greater than the sum of the parts. While numerous scholars have used the syndemic approach to understand health issues, relatively few investigations provide an operational approach to measuring a major element of syndemics: the excess burden of poor health at the population level.

To summarize, Mendenhall and Singer [11] and Gravlee (2020) [22] offer three tenets or core features outlining what constitutes a syndemic, and we have proposed a fourth tenet:

1. Social factors: There are large-scale social forces that precipitate disease clustering, including multigenerational social, economic, and power inequities (e.g., colonialism, enslavement, segregation).
2. Disease clustering (Gravlee [22], refers to this as disease concentration): Two or more diseases cluster together within a population, often described simply as comorbidity or multimorbidity.
3. Interaction: There is interaction either via a biological and/or psychological process between/among the diseases, or interactions across the diseases (biological processes) and the social factors. For example, inflammation is commonly documented in the biological literature, whereas stigma has been reported in anthropology ethnographies. Tsai and co-workers (2017) [23] show that there are different pathways for disease interactions. Sometimes the interactions may not be obvious, and there is not always synergy between/among the diseases.
4. Excess burden: We have added this fourth feature to emphasize that as a result of the three core features above, there is not just simply comorbidity, but the excess burden of poor health and/or diseases is amplified because of the synergy of the diseases, producing “more than the sum of the parts” [22].

**Figure 1:**
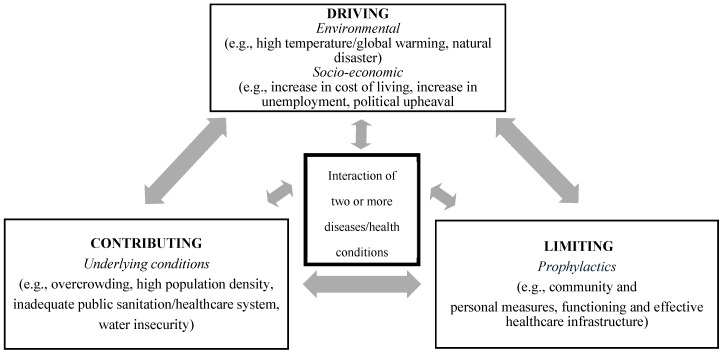
Syndemic paradigm for short-term crisis events, showing the three dimensions.

In addition to the four requisite core features of a syndemic, we propose that there are three factors or dimensions that modulate the expression of a syndemic. These dimensions—contributing, driving, and limiting—can more readily be assessed for short-term crisis events, such as in the case of an epidemic (see Figure 1).

### 1.2. Background on Malta in the 20th Century

#### 1.2.1. Overall Health on the Island

During the early 20th century, Malta’s standard of living was considered low relative to much of Europe. Throughout the study period, the island’s living conditions, sanitation, and health facilities remained persistently inadequate. The working poor constituted the majority of the Maltese population. During the study period, Malta had overcrowding, elevated unemployment rates, large families, and nutritional stress. From 1911 to 1924, the life expectancy at birth for the Maltese was comparatively low at 44 years, in contrast to other European nations [24]). In 1900, the population of Malta had a life expectancy of 40.65 years, while the life expectancy at birth in England and Wales stood at approximately 48.25 years [25]. In 1918, the Maltese exhibited a high infant mortality rate just below 250 per 1000 live births, whereas in another small-scale British colony, Gibraltar, the infant mortality rate was 120 infant deaths per 1000 live births [26]. During this same year, infant mortality rate in England and Wales was much lower at 97.6 per 1000 live births. The diminished survival in Malta during the early 20th century was chiefly attributable to elevated childhood death rates in rural areas [26].

#### 1.2.2. 1918/19 Influenza Pandemic and Early 20th Century Tuberculosis

The influenza epidemic first emerged in Malta at the Cala Frana Seaplane Depot in June 1918, resulting in 31 cases [27]. Between July and August 1918, cases of moderately uncomplicated infection surfaced among the civilian population across six communities on the island: Senglea, Silema, Luca, Tarxien, Paola, Valletta, and Zeitun. According to Dr. Critien, the Principal Medical Officer of Health (PMOH), the herald wave resulted in 34 civilian fatalities [27]. The second wave commenced in mid-August, mostly affecting patients and staff at St. Elmo Hospital, as well as the 1st G.B. Northumberland Fusiliers stationed at Polverista Barracks in Cospicua [27]. The initial documented cases of influenza infection during the second wave occurred within the working classes of Zeitun in September 1918 [27]. The mortality rate for the 1918/19 influenza pandemic in Malta, encompassing both waves, was 3.9 per 1000, comparable to numerous other low mortality rates of influenza deaths recorded in island communities elsewhere during 1918. It was previously observed that on the smaller island of Gozo, children under the age of 5 years transmitted the virus to families, infecting adult females at higher rates relative to adult males [4].

From the early to mid-1900s, tuberculosis mortality rates in the Maltese islands saw a progressive decline. During the First and Second World Wars, the secular trend in tuberculosis mortality was disrupted, resulting in elevated rates of 1.36 deaths per 1000 individuals in 1918, and a decreased rate of 0.84 deaths per 1000 individuals in 1942. Comparable observations of extraordinarily elevated mortality rates during the World Wars have been documented in other studies (see [28,29,30]). The significantly elevated tuberculosis rates in 1917 and 1918 are attributed by most experts to the intersection of the war and the influenza epidemic. Compared to England, tuberculosis mortality rates in Malta were generally lower but exhibited a similar downward trend, except during the Second World War, when Malta’s rates significantly exceeded those of England.

## 2. Materials and Methods

The main objective of this study was to verify whether there was a period of excess burden of mortality associated with the 1918/19 pandemic, as this is a requisite for a syndemic. We also examined whether there was an association of meteorological measures with the potential 1918 syndemic of influenza and respiratory tuberculosis.

### 2.1. Data

To assess life expectancy, mortality information from 1914 to 1923 came from two discrete sources. The Annual Health Reports for the Maltese Islands were used to extract information on cause of death by age bands (under 1, 1–4, 5–9, 10–14, 15–19, 20–24, 25–34, 35–44, 45–54, 55–64, 65–74, 75–79, 80–84, 85 plus) for the years 1914 until 1923, with the exception of the year 1918 and part of 1919 [31,32,33,34,35,36,37,38]. Deaths caused by influenza and respective complications (broncho-pneumonia and pneumonia) and respiratory tuberculosis were not recorded in the health reports during the pandemic of 1918/19. Instead, we used the original government nominal death registers to obtain the count of deaths for each of the age bands for this time period and for the aforementioned diseases [39,40]. The death registry provided details on name, age at death, place of birth, place of residence, and name and surname of parents, whether living or dead. The number of annual births for Malta was extracted from the Annual Health Reports for the same years [31,32,33,34,35,36,37,38,41,42] and was used to adjust the population at risk for under 1 year of age.

To estimate the population at risk for the same age bands as the deaths, we undertook a two-stage method. First, we used the raw census numbers for Malta as reported in the 1911 census [43]. From that baseline, we adjusted the number proportionately using the population size cited for Malta in the Annual Health Report for 1918/19 in the Maltese Islands [41]. The census population numbers for the age categories were used to approximate the population size for 1914 to 1917, which was multiplied by 4 to account for the years of the study period.

The second stage was to correct two common problems found in most population census counts; that is, age misreporting and age heaping. Age misreporting is frequently found in populations with low literacy or low educational levels. Another issue that arises during enumeration is when the respondent over- or under-estimates the age. Finally, the census enumerator may simply report the individual’s age based on his/her physical appearance. Age heaping is another common problem observed in census counts. The problem arises from the respondent preferring to assign an age with a 0 or ending in 5, rather than a precise age. For example, a 69-year-old or a 71-year-old individual may simply round off the age to 70. To correct for age misreporting and age heaping, we used a smoothing method developed by Arriaga [44]. The “reconstructed and smoothed population structure” for Malta in 1918 was entered into the period life table. The same smoothing method was applied to the year 1919. The 1921 census was used to estimate population size for each of the age categories for the years 1920 to 1923, and the estimate was multiplied by 4 to account for the four years of the study [45].

Because of the relatively small geographical size of the island, Malta had a highly localized micro-climate, meaning that meteorological measures were consistent across the island, and were reliable measures to be examined as potential factors involved in the syndemic.

Three meteorological variables in the assessment of the syndemic period (1918/19) relative to prevailing baseline weather conditions (for the years 1906 until 1917) were extracted from the Annual Health Reports on the Maltese Islands [31,32,33,34,41,46,47,48,49,50,51,52,53] The physical properties by month included the following: the absolute minimum and maximum shade temperatures measured in Fahrenheit, the relative humidity given in percent, and the total rainfall amount in inches.

### 2.2. Analysis

To assess secular trends in respiratory morality over the study period, annual tuberculosis death rates for 1908 until 1938 were generated from the total yearly tuberculosis death counts, divided by the population at risk estimated from the 1911 census [43]. These death rates for the two islands were graphed over time.

A temporally defined period of normal or baseline mortality (BM), the mortality experience as determined by life expectancy at birth, was used to measure changes in survivorship during three periods: P_2_, pandemic (1918); P_3_, fallow period (1919); P_4_, post-pandemic period (1920 to 1923). The war years 1914 to 1917 (P_1_) were used to estimate BM.

The Survival Lifetable program (Austin, TX, USA) [54] was utilized to calculate the life table functions (life expectancy at birth (e_(0)_) and probability of dying (q_(x)_)) by employing the period life table methodology, and to evaluate the impact of cause-specific mortality linked to numerous infectious diseases. The period life table provides a cross-sectional view of the mortality and survival experience of a population during a specific year or a group of years. The period life table output was generated using the Survival template by entering the population size counts for each of the age categories and the respective death counts. This was completed for each of the three periods. We also identified and included counts of deaths associated with six distinct cause-specific or disease complexes: (1) influenza pneumonia and bronchopneumonia; (2) respiratory tuberculosis; (3) the diarrhea complex (diarrhea, gastroenteritis, enteritis); (4) the infantile debility complex (marasmus, atrophy, debility); (5) measles; and (6) whooping cough, along with all other causes and residual causes. These disease complexes were chosen because they were known to be common causes of deaths during the study period. The inclusion of the disease complexes allowed for the assessment of the impact of these diseases on life expectancy via the life table function, i.e., the probability of dying (q_(x)_). To quantitatively assess differences in life expectancy across the study periods, two procedures were used. The Z-test, as described by Chiang (1981) [55], was employed to evaluate the importance of life expectancy during the 1918 pandemic, fallow period (1919), and post-pandemic period (1920–23) in comparison to BM (1914–17). The advantages of utilizing the Chiang technique have been detailed in other sources [56,57]. Second, the period life table was complemented by Arriaga’s (1984, 1989) decomposition methodology to identify the age band(s) where mortality differentials existed and to elucidate the contributions of variations in age-specific death rates to changes in life expectancy at birth (across two of the study periods) through both direct and indirect effects [58,59]. The Excel template from Auger et al. (2014) [60] was used to construct the decomposition analysis. The direct impact on life expectancy arises from alterations in life years within a specific age range due to the effects of mortality changes in that age range [61]. The indirect impact refers to the increase in life expectancy attributable to the alterations in the number of survivors at the conclusion of an age interval, resulting from changes in mortality within that interval [61]. The Arriaga method also identified the apportion or contribution of cause-specific differences (from the disease complexes) in life expectancy across two of the study periods. *p*-values indicated whether or not the disease complexes contributed to significant differences in life expectancies. The life table function, showing the probability of dying (q_(x)_) from 1918 influenza and from respiratory tuberculosis during the 1918 influenza pandemic, was graphed to characterize the deaths attributed to each disease by age and sex.

Monthly graphs for the three meteorological measures (temperature, relative humidity, and rainfall) were created for an extended baseline period of 1906 until 1917 to control for the variation that can occur within seasonality. All graphs were created using Statistica (Hamburg, Germany) [62].

## 3. Results

One of the limitations of this study is that we did not have information to assess the syndemic nature of influenza and respiratory tuberculosis at the individual level. We could only establish an indirect relationship between the two diseases. Our retrospective population-based study does not allow for any detailed account of how rainfall and relative humidity affected influenza transmission, other than a possible relationship of changes in behavioural and inter-personnel responses under cooler temperatures and moisture.

**Figure 2:**
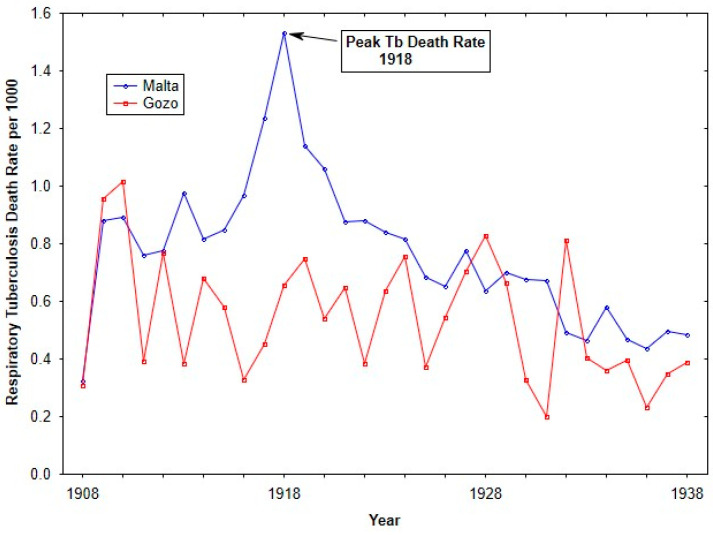
Tuberculosis death rates (per 1000 living) from 1908 to 1938 for Malta and Gozo.

Figure 2 shows Respiratory tuberculosis death rates for the islands of Malta and Gozo from 1908 until 1938. Over the time period, Malta showed higher respiratory tuberculosis rates, in particular during the influenza pandemic. The elevated respiratory tuberculosis mortality rate in 1917 has previously been discussed (see [24]).

The baseline mortality (BM) period was characterized by a life expectancy at birth of 37.92 ± 0.21 years, with childhood infectious disease epidemics contributing to the low life expectancy (see Table 1). With the appearance of the 1918 pandemic, life expectancy underwent a significant decline to 33.26 ± 0.34 years (Z = 10.56, *p* < 0.0001). Even with the continued but muted appearance of influenza in 1919, during this fallow period, there was a dramatic rise in life expectancy to 43.49 years (Z = 17.61, *p* < 0.0001). Following the fallow period in 1919, during the post-pandemic period, there was a significant decrease in life expectancy (Z = 7.08, *p* < 0.0001), yet life expectancy was higher than that of BM, and as such supports that there was a temporary healthy period following the period of excess burden.

**Figure 3:**
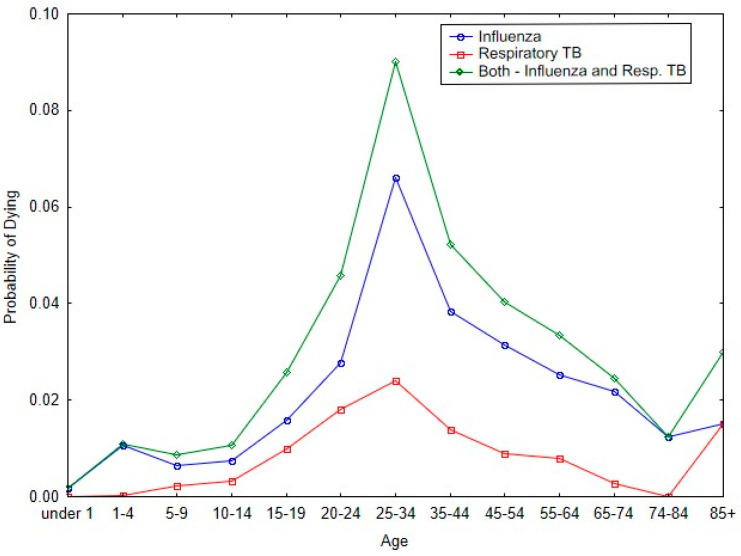
Probability of dying by age categories from 1918/19 influenza and respiratory tuberculosis during the 1918 pandemic.

Figure 3 shows the impact of influenza and respiratory tuberculosis on the probability of dying in the respective age bands for Malta during the 1918 pandemic. The two diseases have similar patterns for the probability of dying with respect to each of the age categories. And for both diseases, the peak probability of dying occurs in the age band of 25 to 34 years of age.

As with Figure 3, Table 2 shows that there was a significant increase in respiratory tuberculosis mortality among reproductive-age individuals during the 1918 influenza period (Z = 19.33, *p* < 0.0001). In contrast, there was not a change in respiratory tuberculosis death among post-reproductive-age individuals (Z = 1.94, ns).

The results of the age decomposition in Table 3 show that the largest contribution to the difference between BM and P_2_ (pandemic) was due to the indirect effect (90 percent). In contrast, the direct effect was about 10 percent and primarily confined to the 25 to 34 age interval. Approximately two-thirds of the indirect effect fell in the age intervals of 15 to 19 years, 20 to 24 years, and 25 to 34 years. The indirect effect represents the life years added to life expectancy because of the change in the number of survivors at the end of each of the respective age intervals.

Table 4 shows that the largest significant differences between BM and P_2_ (pandemic period) occurred because of the 1918/19 pandemic influenza (Δ = 4.052, *p* = 0.001). There was a significant improvement in the probability of dying during the pandemic due to the diarrhea complex (Δ = −0.915, *p* = 0.038). The lack of significant differences in the contribution of respiratory tuberculosis to the differences in life expectancy between the two periods can be attributed to the fact that tuberculosis affected those primarily in their reproductive prime, and the impact of the disease cannot be observed across the span of 86 years (Figure 3; Table 2).

**Figure 4:**
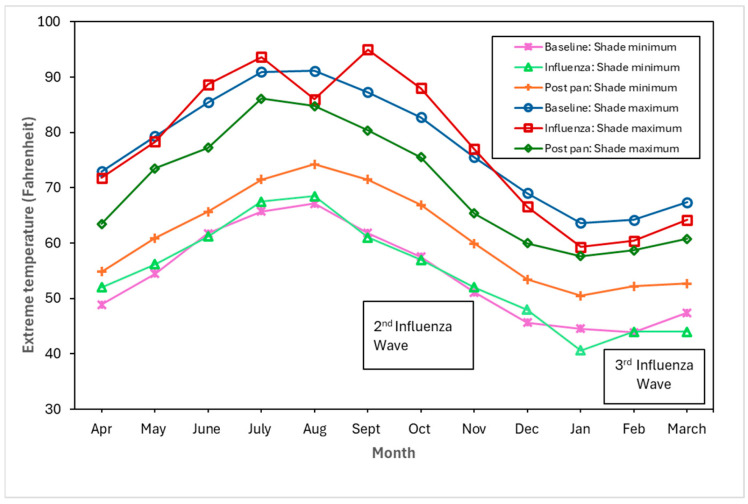
Baseline (1906–1917), pandemic (1918/19), and post-pandemic (1920–1923) minimum shade and maximum shade temperatures.

**Figure 5:**
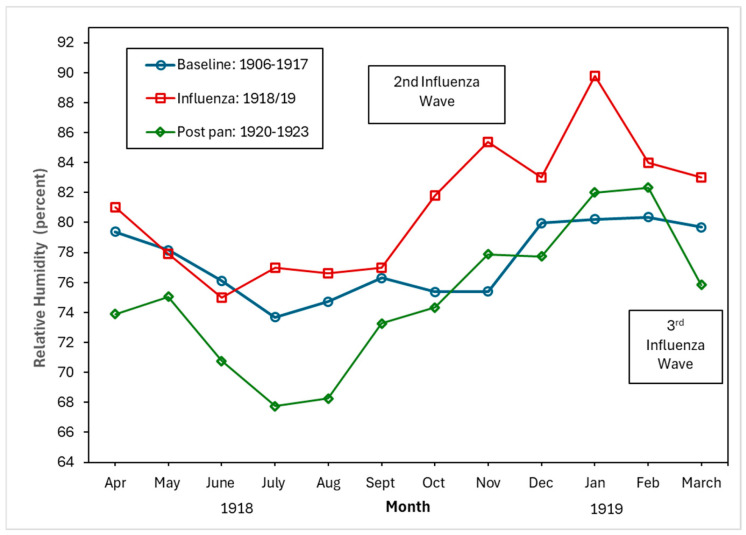
Baseline (1906–1917), pandemic (1918/19), and post-pandemic (1920–1923) relative humidity (percent).

**Figure 6:**
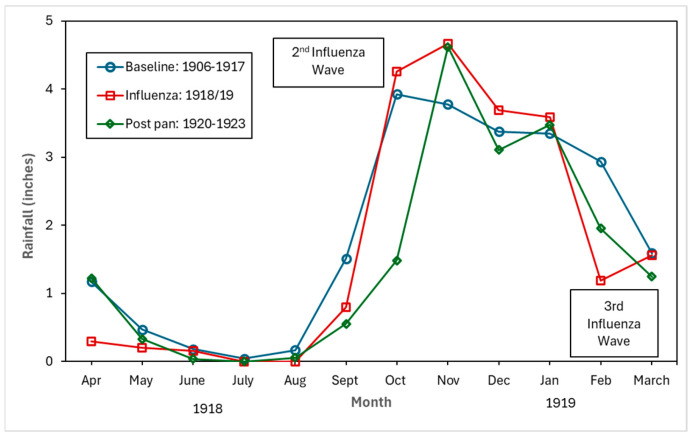
Baseline (1906–1917), pandemic (1918/19), and post-pandemic (1920–1923) rainfall in inches.

Figure 4, Figure 5 and Figure 6 show the monthly patterns of 1918/19 for the absolute maximum and minimum temperatures, relative humidity, and rainfall against the meteorological baseline of 1906 to 1917. A large deviation in higher relative humidity rates during the 1918/19 pandemic relative to the baseline and post-pandemic periods was observed. The relative humidity remained high in the winter months (third wave of the pandemic) (see Figure 5). While largely subjective, it appears that during the second wave of the influenza pandemic (especially in September and October), maximum temperature, relative humidity, and rainfall total (except for September, where BM rainfall total was higher than pandemic total) were higher than in the BM and post-pandemic periods. Not unexpectedly, the pandemic peaked during the relatively cooler temperatures associated with the fall months (see Figure 4). The post-pandemic period had milder temperatures relative to the pandemic and BM periods, with lower maximum and high minimum shade temperatures.

The epidemic-driven syndemic and its complexities can be dissected further, even beyond the quantitative methodology used. A conceptual framework can be used to assess the syndemic built around three dimensions: driving factors, contributing factors, and limiting factors.

We propose the following as driving factors: environment factors such as relative humidity and rainfall; the First World War and the associated deteriorating economy; and the military presence and interconnectedness with the civilian population. These factors facilitated the elevated rates of both 1918/19 influenza and respiratory tuberculosis. Contributing to the driving factors were the underlying living conditions on the island. Lastly, the magnitude of the syndemic was limited by the numerous mitigation strategies, both at the individual and community level, that were put in place early in the pandemic by government officials. These prevented further transmission of and fatalities from influenza and presumably tuberculosis.

## 4. Discussion

Our study validated the existence of a syndemic during the 1918/19 pandemic. The syndemic of 1918/19 led to a period of unusually low mortality patterns. Beyond the diminished number of deaths due to the influenza complex, we can add that the respiratory tuberculosis death rate among adults (15 to 44 years of age) in 1918 fell from 2.398 per 1000 to 1.736 per 1000 living (Z = 3.077, *p* < 0.003). The overall impact was that during the fallow period of 1919, life expectancy at birth increased to 43.49 years from 32.26 years. The life expectancy during the fallow period was also higher than during the war years (37.92 years). In other words, the syndemic period removed the highly vulnerable in the population, corresponding to what scholars who investigate crisis mortality term as short-term mortality replacement followed by a “healthy period”.

Unlike its larger sister island of Malta, Gozo did not experience a syndemic of 1918 influenza with tuberculosis, because on the smaller island, tuberculosis deaths rates remained relatively stable over the early part of the 20th century. According to the CMOH at the time, Dr. Critien, the agrarian lifestyle of the Gozitans contributed to the lower rates of respiratory tuberculosis because they were spending more time outside, in addition to the isolative nature of the island [41].

The syndemic nature of tuberculosis and pandemic influenza in Malta was even apparent to the health officials at the time. When summarizing the pattern of 1918 influenza during the pandemic, Doctor Critien (CMOH) acutely observed that influenza was “…in my opinion, connected with diminished physical resistance of the masses brought about by the war. In many cases, also intercurrent influenzal infection must have given a spurt to *tubercular disease* where it was active and contributed to fan it into actively where it was latent” [41].

Based on the proposed conceptual framework, we propose the following as contributing to, driving, or counteracting/limiting the syndemic:

### 4.1. Contributing Factors: Underlying Living Conditions

Even though there would have been regional variation with regard to the living situations across the island, the general consensus was that the majority of the Maltese were poor. A number of prevailing conditions on the Maltese Islands during our study period created an ideal environment that facilitated the spread of infections, especially frequent childhood epidemics such as measles and whooping cough, as well as endemic diseases such as gastro-enteritis, undulant fever [63], and trachoma [64]. These conditions included the following: first, the standard of housing in most towns and villages was low; second, the houses were small, with overcrowding the norm. For example, in 1911, the average number of persons in a dwelling unit, exclusive of institutions, was 4.84 [43]. Third, family size was large. For women who were married at under 20 years of age, the average number of children was 10, and for women who married between 20 and 25 years of age, the average number of children was 8. Regardless of age at marriage, on average there were 6.51 children per family [65,66]. As such, many children were forced to sleep together in a single room in close contact. Fourth, laid-on water within the household was lacking, and it was often necessary to draw water from public taps and carry it home, thereby limiting personal hygiene and the frequency of laundering. Family members were forced to use the same towels and wash in the same water, such that personal cleanliness was a constant hygiene issue in Maltese households. Fifth, sanitary conditions in and outside the home were abysmal, facilitating the breeding of flies and vermin. Public and household sanitation amenities were poor or virtually absent. In rural settlements, open-air cesspits and latrines were commonplace. Finally, the diet of the lower classes was universally poor: rich in carbohydrates but lacking in fats or meat.

### 4.2. Driving Factors: Environmental

Despite the vast body of literature on the 1918/19 influenza pandemic and the myriads of factors that played a role in the spread of the pathogen (see, for example, [67]), the importance of meteorological factors such as temperature, rainfall, and relative humidity has received scant attention with regard to pandemic influenza. And although there has been a dearth of studies on the relationship between these meteorological factors and respiratory tuberculosis, the few contemporary studies from China show that there is a correlation between high temperatures, high relative humidity, windspeed, and the incidence of pulmonary tuberculosis [68]. However, some studies found a negative correlation between relative humidity and tuberculosis risk, and that there were lags (in days) in the relationship between meteorological factors and the relative risk of tuberculosis cases [69]. While our research offers only a qualitative perspective, it suggests that unlike the cool and dry conditions proposed as a driver of seasonal influenza [67], higher maximum temperatures, relative humidity, and higher rainfall amounts (compared to the baseline) were clearly associated with the second and third influenza waves (for relative humidity only) of the 1918/19 pandemic. In Malta, during the autumn and winter months with cooler temperatures, the lack of central heating meant that residents of a household would crowd together around a stove fueled by paraffin or propane. Increased familial interpersonal contact under these circumstances would increase the probability of droplet pathogen spread.

### 4.3. Driving Factors: Declining Economy During the First World War

The national economy of Malta during the war relied heavily on servicing the Royal Navy. As the war progressed, there was a decline in war-related activities, resulting in an increasing number of men and women who were working at lower wages relative to the years before the war, and a significant number of unemployed individuals. The war effort originally generated economic prosperity for the Maltese, but this wealth was short lived. By 1916, the cost of living had doubled, accompanied by increased unemployment rates, employment insecurity, and food shortages. Furthermore, the prices of food items increased; they were of substandard quality and challenging to obtain, even at elevated costs [70]. For instance, between 1914 and 1918, the prices of high-protein food items (fish, meat, cheese, eggs) soared from 200% to 500% [71]. Bread served as a crucial food staple, primarily providing energy for the impoverished. Throughout the First World War, wheat prices surged as flour supplies diminished markedly, resulting in a threefold increase in bread prices by the war’s conclusion. The severe bread scarcity incited social discontent and a walkout at the Malta Dockyard in May 1917.

### 4.4. Driving Factors: Increasingly Poor Housing Conditions, Overcrowding, and Water Insecurity

Additional evidence of the precarious living conditions was the scarcity of available and new dwellings, a deficiency that undoubtedly exacerbated congestion since family sizes in Malta persisted unabated. We contend that household security was undermined not only by increasing unemployment rates, low wages, and precarious employment, but also by the decline in new housing construction throughout the war years, which intensified the prevailing overcrowding issue. The stagnation of housing was apparent in 1915, as the number of houses constructed decreased from 152 in Malta to 17 by 1920–1921. This was exactly the time when respiratory tuberculosis rates increased. Improvements in living conditions occurred only in 1922, with the construction of 5311 dwellings in Malta between 1922 and 1933 [72]. It would appear that there was a decline in population density between 1911 and 1921 (the two census periods). In 1911 (pre-pandemic), population density stood at 1815 persons per square mile, which dipped to 1778 persons per square mile in 1921 (post-pandemic). This small decline, however, was most likely a reflection of the emigration of the population that occurred after the armistice in November 1918, when emigration by the government was encouraged to alleviate overcrowding on the island [73]. By 1931, population density surged to 2016 persons per square mile. Further, overcrowding at the household level (measured by when there are more than two people living in one room in a tenement of fewer than five rooms) was relatively high during the pre-pandemic period in 1911 at 20.9 percent, dropping slightly to 19.07 percent of families being in overcrowded homes in 1921. In keeping with government officials’ comments on improvements in living conditions after 1921, household overcrowding dropped to 16.5 percent by 1931 [43,45,74] (Government of Malta: 1912, 1922, 1932). Overcrowding, unemployment, and elevated prices of food and other essentials compromised overall health, while the lack of public welfare for the impoverished further deteriorated the well-being of the Maltese working class.

The Maltese have faced challenges with securing adequate accommodation throughout the centuries, spanning from cave dwellings to present housing. Acquiring accommodation that was spacious, ventilated, well drained, clean, and possessed vital water resources was very important in maintaining the well-being of the Maltese. Access to a plentiful supply of potable water is arguably the most critical component for both cleanliness and for drinking purposes. Due to the size and geography of the islands, water supply was critical for the maintenance of a sanitary home environment, the cleansing of cooking and eating materials, and the washing of clothing. This was particularly important during the long hot summer months, where a shortage of rainwater was a constant source of anxiety and stress. The risk of contracting enteropathogens from their immediate surroundings, given the unhygienic state at the household level, was a constant health risk during the study period.

### 4.5. Limiting Factors: 1918/19 Influenza Mitigation Strategies

The first mitigation measure was introduced on 9 September 1918, and was printed in the newspaper. The notice included information on droplet transmission and handkerchief use, and emphasized the importance of isolating the sick, as well as fresh air and light for destroying the virus. It instructed readers to avoid gatherings in crowded places. The PMOH, Dr. Critien, recommended that the leaflet be translated into Italian and Maltese [75].

By September 16th, both individual and general prophylaxes had been implemented. At the individual level, there was to be isolation at home or removal to the Manoel Infectious Diseases Hospital for severe cases, and in cases complicated by pneumonia or bronco-pneumonia, in addition to the disinfection of rooms, bedding, and linen.

In cases of influenza that originated in other hospitals, charitable institutions, prisons, ships in harbor, and/or in cases without proper care and accommodation, it was recommended that patients be transported to the Manoel Infectious Diseases Hospital [75].

At the general or community level, the following measures were put in place:

1. Prevention of overcrowding in public places, cinemas, theatres, and other places of amusement; cleanliness, aeration, and disinfection;
2. Disinfection of public places with large amounts of people, such as railway carriages and ferryboats;
3. Reduction in visitations to hospitals and other charitable institutions; discontinuance of pawning off clothes, etc.;
4. Closure of government schools;
5. Increased visiting of dwellings, etc., by sanitary inspectors;
6. Temporary surveillance of all arrivals from abroad and disinfection of personal belongings in certain cases.

For other prophylactic measures that were implemented during the pandemic, please see Supplementary Table S1. Table S1 details the 1918/19 influenza pandemic mitigation strategies in Malta.

## 5. Conclusions

Following a method to quantitively operationalize short-term syndemics previously proposed by two of the authors [18], we demonstrated that there was a syndemic relationship during the 1918/19 influenza pandemic of the influenza virus with respiratory tuberculosis. Life expectancy during the pandemic significantly dropped (LE = 33.26 ± 0.340) compared to the pre-pandemic and war years (LE = 37.92 ± 0.209; Z = 10.56; *p* < 0.0001). Further, in 1919, life expectancy exceeded the pandemic life expectancy (LE = 43.49 ± 0.431; Z = 17.61; *p* < 0.0001) and surpassed levels observed during the study period, showing evidence of the harvest effect followed by a “healthy period”. The fact that the respiratory tuberculosis death rate among those in their reproductive years significantly rose during the pandemic from 1.60 to 2.40 per 1000 living (Z = 19.32; *p* < 0.00010), and significantly declined to 1.74 per 100 living in 1919 (Z = 3.08; *p* < 0.003), is indicative of a crisis event and a syndemic with influenza. We propose that when there is a potential syndemic resulting from a short-term event (such as an epidemic), it would be beneficial to extend our reproducible method to also include an assessment of the dimensions of the syndemic contributing, driving, and limiting factors. In our study of the 1918/19 pandemic in Malta, the contributing factors were the existing inadequate living conditions that were conducive to the spread of infectious diseases, especially childhood infections.

We qualitatively observed environmental factors such as ambient temperature, relative humidity, and rainfall as potentially driving the syndemic. Another driver of the syndemic was the economy associated with the First World War. The exacerbation of living conditions such as overcrowding was an additional driving factor of the syndemic. Finally, the early implementation of effective mitigation measures, despite the lack of knowledge on the transmission of the virus, such as limiting mass gatherings and disinfecting public buildings and institutions, would have worked to limit the extent of the interaction of the two diseases, reducing the transmission of both influenza and tuberculosis.

Our framework to conceptualize the syndemic potential during the 1918 influenza pandemic in Malta contributes to the study of syndemics beyond the small former British colony and beyond studies of past epidemics and pandemics. This framework, which is grounded in crisis mortality and the harvesting effect, can easily be applied to the assessment of other short-term novel events. Further, to capture the factors involved in the syndemic, exploring contributing, driving, and limiting dimensions is useful for deconstructing the components involved in situations where syndemics occur over a limited period of time.

## Figures and Tables

**Table 1:** Life expectancy at birth during the four periods from 1914 to 1923 in Malta.

Period	Year	Epidemics	LE (ex0)	SE	Z Score	*p*-Value
War (P1)	1914–17	Measles, whooping cough	37.915	0.209		
Pandemic (P2)	1918	Influenza—severe	33.264	0.340	10.56	<0.0001
Fallow period (P3)	1919	Influenza—mild	43.489	0.431	17.61	<0.0001
Post-pandemic (P4)	1920–23	Influenza—slight	40.093	0.212	7.08	<0.0001

**Table 2:** Respiratory tuberculosis death rates (per 1000 living) in Malta for the four time periods.

Age Group	War (P_1_)	1918 (P_2_)	1919 (P_3_)	Post-Pandemic (P_4_)
15–44 (reproductive ages)	1.604	2.398	1.736	1.339
45 plus (post-reproductive ages)	0.525	0.823	0.601	0.626

Z-score tests: Reproductive period: P_1_ vs. P_2_, Z = 19.323, *p* < 0.0001; P_2_ vs. P_3,_ Z = 3.077, *p* < 0.003; P_2_ vs. P_4_, Z = 6.104. *p* < 0.0001. Post-reproductive period: P_1_ vs. P_2_, Z = 1.943, ns; P_2_ vs. P_3_, Z = 1.046, ns; P_2_ vs. P_4_, Z = 1.239; ns.

**Table 3:** Age-specific effects on LE differences between war period (1914–1917) and 1918 pandemic period (1918), Malta.

Age	Total Effect	Percent Direct	Percent Indirect
Under 1	−0.17	−0.01	3.65
1 to 4	−0.04	0.04	0.83
5 to 9	−0.13	0.16	2.74
10 to 14	−0.34	0.43	6.91
15 to 19	−1.10	1.47	22.23
20 to 24	−0.75	1.07	15.01
25 to 34	−1.56	4.82	28.75
35 to 44	−0.38	1.48	6.75
45 to 54	−0.30	1.55	4.91
55 to 64	0.17	−1.24	−2.44
65 to 74	−0.09	0.94	1.02
75 to 84	0.05	−0.72	−0.31
85 plus	0.00	−0.06	0.00
Total	−4.65	9.94	90.06

**Table 4:** Apportion of cause-specific differences in life expectancy at ages 0 to 85 years between P_1_ (war period, 1914–1917) and P_2_ (pandemic period, 1918) in Malta.

Cause	LE Difference (Δ)	Probability (*p*-Value)
Influenza	4.052	0. 001
Respiratory tuberculosis	0.592	0.18
Diarrhea, enteritis and gastro-enteritis	−0.915	0.038
Infantile atrophy, debility, and marasmus	0.744	0.092
Measles	−0.461	0.296
Whooping cough	−0.26	0.557
All other causes	0.913	0.039
Residual (distributed among causes)		−0.001
Total	4.664	

## Data Availability

Data will be made available upon request.

## Supplementary Materials

The following supporting information can be downloaded at: https://www.mdpi.com/article/10.3390/tropicalmed10060149/s1, Table S1: Mitigation strategies during 1918/19 influenza pandemic in Malta.

## Abbreviations

The following abbreviations are used in this manuscript:

TB	Tuberculosis
BM	Baseline mortality
