# Supplementary material for: Assessing the 1918/19 Pandemic Influenza and Respiratory Tuberculosis Interaction in Malta: Operationalizing a Syndemic During a Crisis Event
Source: Trop Med Infect Dis. 2025 May 24;10(6):149. doi: 10.3390/tropicalmed10060149 (PMC12197708; doi:10.3390/tropicalmed10060149)
Supplement: Supplementary file 1 [file tropicalmed-10-00149-s001.zip › tropicalmed-3443810-supplementary.pdf]

Supplementary Materails

Table S1. Mitigation strategies during 1918/19 influenza pandemic in Malta.

Date	Mitigation strategy
Sept 9th 1918	The leaflet on *Influenza and how it may be avoided,* published by the Public Health Department is printed in the newspaper. The notice included information on droplet transmission and handkerchief use; and emphasized the importance of isolation of sick, and fresh air and light for destroying the virus; and instructed to avoid gatherings in crowded places.<br>The C. G. M. O. & Superintendent (Dr. A. Critien), recommends that the leaflet is translated into Italian and Maltese. He also requested that that the Government Elementary schools at Zeitun be not reopened for the present.
Sept 14th	The leaflet on *Prevention of the Spread of Influenza*, is published for the Commanding Units stationed in Malta. The content was similar to the leaflet published for the civilians
Sept 16th	In addition to *Influenza and how it may be avoided*, the C. G. M. O. & Superintendent, described numerous other measures that have been implemented:<br>Individual prophylaxis:-<br>Isolation at home or removal to Manoel Infectious Diseases Hospital of severe cases, and of cases complicated by pneumonia of Bronco-pneumonia; disinfection of rooms, bedding and linen of same.<br>Removal to Manoel infectious Diseases Hospital of influenza cases developing in other hospitals and charitable institutions, prisons, ships in harbor and, as much as possible or cases without proper care and accommodation.<br>General prophylaxis:<br>1. prevention of overcrowding in Public places, cinemas, theatres and other places of amusement; cleanliness, aeration and disinfection of same. 2. disinfection of public places with large concourse of people, railway carriages and ferryboats. 3. deduction of visiting in hospitals and other charitable institution; discontinuance of pawning off clothes, etc. 4. closure of government schools. 5. increased visiting of dwellings etc. by sanitary inspectors, and 6. temporary surveillance of all arrivals from abroad and disinfection of personal belongings in certain cases
Sept 18th & 19th	The L. G. inquired as to whether cinemas should be closed during the epidemic. The Acting C. G. M. O. (Dr. A. Bernard), replied that as per the *Influenza and how it may be avoided* leaflet*,* the Health Dept has been recommending avoiding crowded places, and he does not believe that theatres are places where is "dissemination of the epidemic." He has, however, ordered that that cinemas be disinfected every morning and impressed on proprietors the importance of well ventilated and not overcrowded establishments during performances.
Sept 19th	Hon. G. Vassallo, Esq. sent an original request to the L.G. to temporarily close the cinemas, however, due to an oversight in the sender information, there was not a reply until the 23rd. It does not appear that cinemas were closed during the epidemic.
Sept 20th	In addition to the measures outlined on the 16th, the Acting C. G. M. O. mentioned a couple other precautionary measures to date: disinfection of premises and belongings after death of patient or his removal to isolation at Manoel Hospital or Apartment No. 9 at the Lazaretto; surveillance, and, in certain cases, disinfection of contacts; recommendation to disinfect daily the Courts of law
Sept 20th & 21st	The Acting C. G. M. O. put in a request that the military authorities be approached as to whether they could lend the use of a motor ambulance on the government paying for petrol consumed, etc. The ambulance would serve for carrying patients to hospital, bedding etc. to the disinfecting station and it could also be used by the Medical Officers of this Department for urgent visits. E. Bonavia asked on the 21st for the expenses incurred by Public Health dept. could be refunded by the C. govt.<br>The motor car was provided on the Sept 21st
Sept 20th	The Acting C. G. M. O. requested an increase pay for Sanitary Inspectors of at least 30%, because, "under present circumstances their duties assumed greater importance and are very arduous."
Sept. 23rd	The Acting C. G. M. O. & Superintendent wrote to the Lieutenant-Governor Government Schools at Gozo be kept closed until further notice, because cases of Influenza have now occurred in Gozo
Sept 25th	The Lieutenant-Governor inquired whether cinemas and schools should be disinfected, and if it is practical to ask the same of churches. The Acting C.G.M.O responded that the government schools are closed, and that private schools will not be in full attendance until October. He "recommended thorough ventilation, cleansing and spraying with disinfectant lotion. Similar recommendations have been made for cinemas."<br>G.P. Archbishop, following the recommendations of the Acting C.G.M.O, issued a circular to two rectors of churches instructing them to keep place is fully aired and frames of confessionals are to be purified by disinfectants daily. Sprinkling of floors with disinfectants has also been recommended.
Sept 27th & 28th	The C.G.M.O. & Superintendent wrote to the Honourable the Crown Advocate, on Sept 27th, that enacted by order of His Excellency the Governor (Methuen), article 38 of the Fourth Sanitary Ordinance, 1908, as means to give legal force to the cleaning and disinfecting of cinemas (check that it is just cinemas)<br>The draft is approved by the Lieutenant-Governor (Sir W. C. F. Robertson) on Sept 28th-
Oct. 24th	The Lieutenant-Governor, granted the temporary allowance of 30% on the salary of Sanitary Inspectors and that a special warrant for £100 to be issued covering expenditure until January
Oct 31st	The C. G. M. O. & Superintendent stated that there is no longer a permanent need for the motor ambulance on loan by the Military Authorities because number of severe cases of influenza is on the decrease.

Nov 22nd & Nov 23rd	The C. G. M. O. & Superintendent recommend that Government Elementary schools in Malta be reopened as of the 2nd of December. The Director of Elementary Schools (D.E.S.), noted on the following day that, the C. G. M. O. has informed him each school will be inspected on opening to ensure proper disinfection and that "doubtful cases are not allowed to attend."
Nov 25th	The C. G. M. O. & Superintendent, described measures that teachers should take to minimize he spread of influenza within schools, once they reopen. The document was seen by the D.E.S.<br>1. Teachers in charge of classes should make an inspection of all the children attending, every morning before the beginning of class.<br>2. Teachers in charge should make a list of<br>a) children who are evidently suffering from cold or cough. b) children who are who on inquiry at the time of inspection are found to be coming from houses where there is a case of illness. c) absentees.<br>3. Children under a should be sent home at once.<br>4. list will be called for daily by the sanitary inspector in charge.<br>5. Directions as to children named in list Stingray admitted or excluded etc. will be sent to headteacher as soon as possible.<br>Teachers and Department teachers suffering from a Cold War from influenza should not attend.
Nov 27th	The C. G. M. O. & Superintendent, described in a circular the steps that Sanitary inspectors would take prevent influenza once schools have reopened.<br>Sanitary inspectors were instructed to call daily at 10:00 AM from 2nd proximo on school days at Government Elementary School of their district where they will require list of children as follows:-<br>a) Children listed as suffering from a cold or cough {See parents or persons having custody of the child and inform them that they should obtain medical certificate from their private doctor or D. M O. If child is certified as not suffering from influenza or other notifiable disease he may be reemitted to school.<br>b) Children listed as coming from houses where disease exists {Call at child's address and inquire; if illness is likely to be influenza or its complications, ask for name of doctor attending and note information on list. If illness is some ordinary complaint. Note on list.<br>c) Children returned as usual {If absence is due to illness act as (a), second para of (b).<br>6. Lists, with remarks as above, should be sent in at once to the M. O. H. c/c/ of District
Dec 7th	The C. G. M. O. & Superintendent requested that the Elementary Schools and Secondary School, Gozo, with the exception of the Elementary School at Caccia be reopened. The Lieutenant-Governor approved the request on the same day.
Dec 9th	The Lieutenant-Governor approve that the Secondary School Gozo to be reopened the following day, the 10th at 9 a.m. There are only nine school days to the end of the term. It was suggested that no fees be charged for the term. The 2nd will begin normally on Jan. 2nd.
Jan 4th &and 7th 1919	On Jan 4th, The C. G. M. O. & Superintendent reported to the Lieutenant-Governor, that the Elementary School at Caccia, Gozo may be reopened from 7th.
Jan 7th & 8th	After receiving permission the previous day from the Lieutenant-Governor, the C. G. M. O. & Superintendent, wrote to the Editor of the newspaper on Jan 8th requesting that he republish the enclosed leaflet, *Influenza and how it may be avoided*, because "There is a slight recrudescence of the disease and it is well that the public be warned not to slacken in taking individual precautions, especially as a winter months have always have been always a period of danger."
Jan 18th	The District 1 Medical Officer (Dr. A Pullicino), wrote to the Lieutenant-Governor, regarding the reoccurrence of influenza. He noted that: "The Sanitary Authorities have taken a wise step in republishing the preventative measures recommended in September last, but we think it as well to advise the government to adopt without delay more energetic measures calculated to effectively safeguard public health."
Jan 28th	The Lieutenant-Governor and Chief Secretary informed the District 1 Medical Officer that the government would be open to suggestions of "more energetic measures to combat the disease."
Feb 15th	Hon A. Dalli (The Council office), wrote about his concerns of overcrowding during the dances associated with the Veglione Festival, and recommended the establishments which will be hosting the ball, should disinfect and ensure adequate ventilation as well a limit the maximum number of admissions to "avoid dangerous and anti-hygienic overcrowding, the observance of which might be controlled by stamping admission tickets with the Public Health stamp."
Feb 19th & Feb 20th	The council of health decided, that a warning to the public be issued by the public health Department and that steps to be taken to ensure proper Infection and ventilation of premises on which public balls and held during carnivals.<br>The C. G. M. O. & Superintendent stated instructions are to be inserted in permits issued by the Police to persons applying to hold dances or other entertainments: "That all parts of the premises where dances or other entertainments are held be kept perfectly clean and thoroughly ventilated to the satisfaction of the Superintendent of public health during the entertainment…That all parts of premises where such entertainments are held be disinfected at least once daily to the satisfaction of the Superintendent of public health.<br>Non compliance with the above will entailed immediate closure of any place to which this permit applies and holders of licenses will be prosecuted. These provisions are contained in Government Notice No. 361 of 4th October 1918 but it is well to bring them to the immediate notice application applicants."
March 5th	C. G. M. O. & the Superintendent recommended the closure of village schools in Melleha on March 5th 1919
March 12th	The Lieutenant-Governor wrote that due to the prevalence of influenza, it has made it necessary for the C.G.M..O to take further precautionary measures.

	He has been encouraging the C. G. M. O. & the Superintendent to take the necessary immediate actions that will be required to stop the spread of the disease, regardless of financial concerns. Any delay of measures may in fact increase expenditure at a later date. He predicts it will be probable that all schools will have to be closed.
March 13th	The Comptroller of Charitable institutions asked the Lieutenant Governor for an increase in traveling expenses on not exceeding the rate of 5s/- a day, so that the District Medical Officer for Sliema can visit patients within the district. The officer has to travel on foot practically the whole day, often retraversing the distance is already done and after whole days hard work is also liable to be called during the night. This same concession was granted during October 1918.
March 15th	Approval was granted by the Lieutenant-Governor to immediately print 10,000 copies of the notice informing parents that children will require a medical certificate before attending schools after being sick with influenza. The notice was written by the C. G. M. O. & the Superintendent (The Public Health Dept). The notice stated:<br>The attention of parents or other persons having the care of children, as well as of teachers or persons in charge of schools, is called to the provisions of the law under which children who are or have been suffering from an infectious disease, e. g. Influenza, or who reside in a house where such disease exists are not allowed to attend without a medical certificate to the effect that they are free from disease and infection.<br>Parents, teachers or other persons in charge of children or schools are liable to prosecution and to a penalty not exceeding £10.
March 18th	The Director of Elementary Schools (D. E.S.), informed the Lieutenant-Governor, that following the instructions of the C. G. M. O., he issued the closing of Notabile, Zebbug, Chircop, and Zabbar Government Elementary Schools.
March 19th	The C. G. M. O. & the Superintendent, wrote a summary of precautionary measures that are in place to date, and described whether other measures are warranted.<br>For cinemas and theatres, the measures of: preventing of overcrowding, increasing maintaining ventilation; and ensuring cleanliness and disinfection of floors are being enforced. Dr Critien *does not see sufficient reasons for their closure*, however, because they are under sanitary control, they are not responsible for spreading the disease to any appreciable degree, and they are very little attended. He recommends that there should be an interval of say 20 to 30 mins between one show and the other to allow for a complete renewal of air, to decrease the risk of transmission.<br>For churches, Dr. Critien and Dr. Bernard, have suggested to Monsignor Portelli on many occasions, the importance of increasing and maintaining ventilation, and ensuring cleanliness and disinfection floors. He further suggested that his Grace the archbishop renew and enforce his orders for the continuous ventilation, daily washing floors of churches; purification of confessional gratings, disinfection of all corners, and limitation of the number of persons entering houses with the Viaticum, the shortening as much as possible of all religious functions. It will also be very beneficial if parish priests were instructed, by his Grace, to warn their parishioners to refrain from attending religious services as soon as they present influenza like symptoms and *to close schools of religious instruction*.<br>The Governor (Third Baron Methuen) sent a minute regarding Dr. A Critien's instructions on preventing the spread of the disease to the His Grace The Archbishop, Bishop of Malta.<br>To prevent infection via inhalation of air "laden with particles of dust freshly contaminated with mouth, nose, and bronchial secretions", there is currently an order for the daily washing and disinfection of certain streets in places in Valletta and Sliema, where people are used to loiter and congregate, and generally the better scavenging of all populated centres.<br>To prevent infection via contaminated objects, such as glasses, cups, spoons, Dr. Critien sough the approval of the Governor to issue regulations that keepers of all coffee shops, public houses, water stalls, restaurants, clubs and hotels should disinfect all crockery immediately after use by immersion in a suitable disinfectant.
March 28th	The C. G. M. O. & the Superintendent revised his opinion about disinfecting public eateries, stating that the lack of suitable disinfectant makes it impractical to enforce regulation.
April 1st	The Rector of the University (E. Margo) to The Lieutenant Governor & Chief Secretary, stating that that all the Schools of the University have been reopened this day, because the C. G.M.O. deems that it is no longer necessary for the University to remain close.
April 1st	The C. G. M. O. & the Superintendent stated that the Sanitary Inspector who is no longer needed by the Military has been re-employed as Extra Sanitary Inspector for "duty at Cottonera where Influenza is very prevalent."
April 3rd	The C. G. M. O. & the Superintendent, recommended to the Lieutenant Governor, that the Government Elementary School in Melleha be reopened on the following Monday
April 10th	The C. G. M. O. & the Superintendent, wrote to the Lieutenant-Governor, that he has arranged with the D.E. S. that all schools be opened in Malta after Easter, (which is in a few days) so that schools can be thoroughly washed, and then disinfected by the Health Department before reopening.<br>He noted that all schools in Gozo, with the exception of schools at Nadur and Xeuchia, have been reopened, including Secondary school. As soon as influenza will abate at Nadur and Xeuchia, the schools in these villages will also be reopened.
April 17th	C. G. M. O. & the Superintendent, requested from the Lieutenant-Governor, the continuation of employment of five extra sanitary inspectors at 3/4 per diem each, "until such time as their service are considered necessary compatibility with the fluctuation of the epidemic of influenza." He noted that the services of two Inspectors will be dispensed with on the 19th.
May 8th	Pay increase for the District Medical Officer for Sliema, has ceased.
